# The effect of different depth planes during a manual tracking task in three-dimensional virtual reality space

**DOI:** 10.1038/s41598-023-48869-w

**Published:** 2023-12-06

**Authors:** Hyeonseok Kim, Yasuharu Koike, Woong Choi, Jongho Lee

**Affiliations:** 1 Swartz Center for Computational Neuroscience, Institute for Neural Computation, University of California San Diego, La Jolla, CA 92093 USA; 2 Institute of Innovative Research, Tokyo Institute of Technology, Yokohama, 226-8503 Japan; 3 College of ICT Construction & Welfare Convergence, Kangnam University, Yongin, 16979 Republic of Korea; 4 Department of Clinical Engineering, Komatsu University, Komatsu, 923-0961 Japan

**Keywords:** Perception, Human behaviour

## Abstract

Unlike ballistic arm movements such as reaching, the contribution of depth information to the performance of manual tracking movements is unclear. Thus, to understand how the brain handles information, we investigated how a required movement along the depth axis would affect behavioral tracking performance, postulating that it would be affected by the amount of depth movement. We designed a visually guided planar tracking task that requires movement on three planes with different depths: a fronto-parallel plane called ROT (0), a sagittal plane called ROT (90), and a plane rotated by 45° with respect to the sagittal plane called ROT (45). Fifteen participants performed a circular manual tracking task under binocular and monocular visions in a three-dimensional (3D) virtual reality space. As a result, under binocular vision, ROT (90), which required the largest depth movement among the tasks, showed the greatest error in 3D. Similarly, the errors (deviation from the target path) on the depth axis revealed significant differences among the tasks. Under monocular vision, significant differences in errors were observed only on the lateral axis. Moreover, we observed that the errors in the lateral and depth axes were proportional to the required movement on these axes under binocular vision and confirmed that the required depth movement under binocular vision determined depth error independent of the other axes. This finding implies that the brain may independently process binocular vision information on each axis. Meanwhile, the required depth movement under monocular vision was independent of performance along the depth axis, indicating an intractable behavior. Our findings highlight the importance of handling depth movement, especially when a virtual reality situation, involving tracking tasks, is generated.

## Introduction

From a motor control viewpoint, manual tracking movements involve both feedback and feedforward control^1^. The extent to which the brain relies on feedforward control for successful performance depends on several factors, including age^2^, disorders^3^, target acceleration^4^, and target speed^5^, involving sub-movements or intermittency^6–8^. Unlike reaching movements, which are likely carried out by initial feedforward control transitioning into feedback control when closer to a target, visually guided tracking tasks require reliance on perpetual processing of spatial information involving visual feedback. Additionally, many manual tracking tasks have been performed in two-dimensional space^9–13^. Those performed in three-dimensional (3D) space have not focused on the effect of depth, as the required trajectory has been somewhat sophisticated^14^. However, depth information of the target should be investigated to understand how the brain plans and generates motor commands to perform tracking movements, as depth perception requires intricate computation in the brain^15^. In other words, the extent to which depth information contributes to performing a manual tracking movement in 3D space remains to be elucidated.

Depth perception is associated with considerable information processing in the brain. Under binocular vision, binocular disparity has been regarded as an important factor in depth perception^16^; therefore, several models have been suggested to explain how the brain can exploit this information^17^. Binocular vision is also related to binocular fusion, which can influence depth perception^18^. Such information is only available under binocular vision and could have advantages in motor control^19^. Relevant brain activity has also been reported, including activity in the dorsal pathway for depth perception by fusing relative motion and binocular disparity^20^, neuronal activity associated with depth based on motion parallax^21^, and brain activity in the posterior parietal cortex and dorsal stream associated with the kinetic depth effect^22^. Models explaining depth perception under monocular vision have also been suggested^23^. Since depth perception depends on various types of available information^24^, it is important to investigate how this information acts at the performance level.

Thus, we focused on the effect of depth information in a visually guided tracking task, which could be useful for learning complex motor skills, especially in a 3D virtual reality (VR) space. 3D interactions have been conducted in situations implemented by different systems, including infrared imaging^25^, augmented reality^26^, and tablet personal computers^27^. We adopted a VR environment to create a situation with a target that spatially corresponded to real-world coordinates for a realistic experience^28^. In VR space, a visual target is completely under our control, permitting the tracer to overlap the target, unlike in the real world, where construction of the environment necessitates a physical entity circularly moving at a constant speed.

If depth information influences tracking performance in a complicated manner, it should be considered when designing training programs for facilitating motor skills through visually guided tracking tasks in 3D VR space. Although a significant difference in tracking performance between the fronto-parallel plane and sagittal plane was reported in terms of an error on the lateral axis^28^, the contribution of depth information to the performance of the task was not explained.

This study aimed to investigate how required movement along the depth axis would influence 3D tracking performance, postulating that the amount of depth movement would affect performance. We designed a visually guided planar tracking task that requires movement on three planes with different depth spaces: a fronto-parallel plane, a sagittal plane, and a plane rotated by 45° with respect to the sagittal plane. In particular, tasks on the fronto-parallel and sagittal planes require movement on two independent axes in the eye-centered coordinate system. However, the plane rotated by 45° with respect to the sagittal plane requires the brain to process three-dimensional information. By adopting this plane, we could determine whether depth information interferes with information processing on the other axes in behavioral performance, regardless of the fundamental processing of the brain. In order to understand the comprehensive effect depth information has on 3D tracking performance, we experimented under two vision environments (binocular and monocular visions) because this category is involved in processing depth information within the brain and can impact tracking performance.

## Methods

### Participants

Fifteen male participants were recruited for this experiment. Table 1 presents the demographic information of the participants. Their ages’ mean and standard deviation (SD) were 20.1 and 0.6, respectively. Two participants were left-handed, and the others were right-handed. All participants provided written informed consent before the experiment. This study was approved by the Institutional Review Board of National Institute of Technology, Gunma College, and conducted in accordance with the Declaration of Helsinki.

**Table 1:** Demographic information of the participants.

Participant	Age	Sex	Handedness
1	20	M	Left
2	20	M	Right
3	20	M	Right
4	20	M	Right
5	20	M	Right
6	19	M	Right
7	21	M	Right
8	21	M	Right
9	19	M	Right
10	21	M	Right
11	20	M	Right
12	20	M	Right
13	20	M	Right
14	21	M	Right
15	20	M	Left

### Experimental apparatus

**Figure 1:**
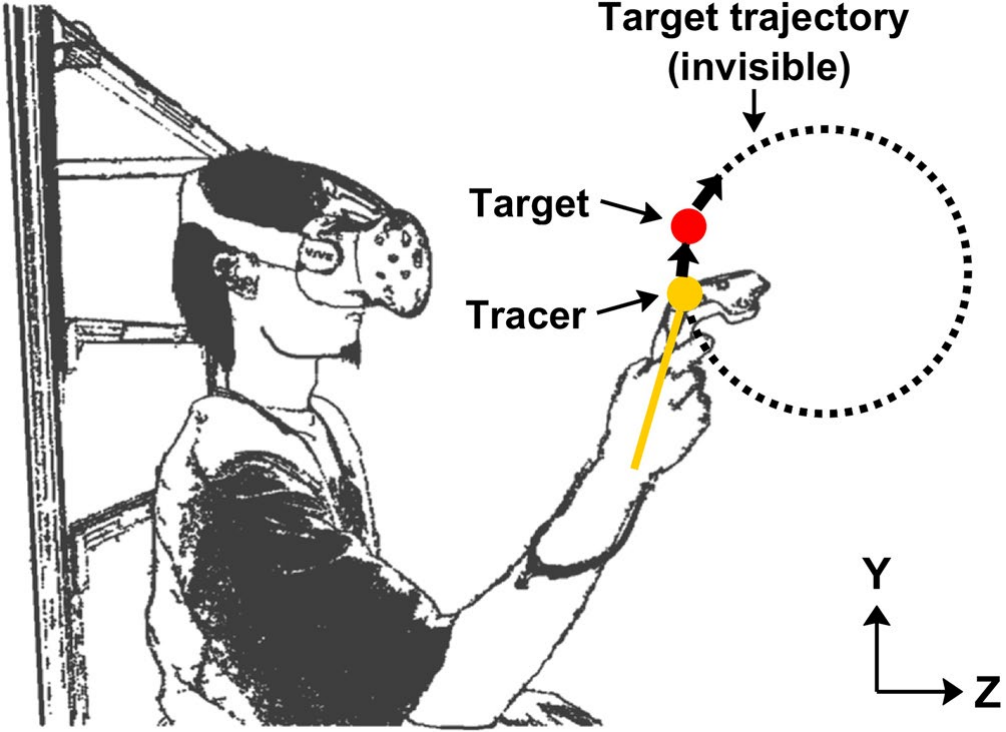
Experimental setup. Participants sat and manipulated a VIVE controller with their dominant hand to move the tracer during the experiment. A red sphere with a diameter of 3 cm was presented as a target, and a yellow sphere with a diameter of 2 cm was presented for the end of the tracer in the VR space. The length of the pole was 20 cm. The actual presented color of the pole in the VR space was white. The target trajectory was invisible in the actual experiment. The diameter of the target trajectory was 30 cm. The X, Y, and Z axes correspond to the lateral, vertical, and depth dimensions. This figure illustrates an image of the YZ plane, that is, the sagittal plane.

In the experiment, participants were asked to perform a visually guided tracking task^28^ with HTC VIVE (HTC Corporation, Taipei, Taiwan) in an immersive 3D VR space, which was implemented using Unity software (Unity Technologies, San Francisco, CA, USA). During the experiment, the participants wore a VIVE head-mounted display (HMD) capable of a refresh rate of 90 Hz, which was used for objects in the VR space. The HMD offered a 110-degree field of view with a 2880 × 1600 pixels resolution. The HTC VIVE system adopts the Lighthouse tracking system^29^ with a position precision of 2 mm. The position of the hand holding the controller and its inclination were visualized with a pole parallel to the controller that had a sphere at one end to indicate the hand position in the 3D VR space, as shown in Fig. 1, so that the participants could keep track of a target moving at a constant speed by controlling the tracer. A red sphere with a diameter of 3 cm was presented as the target, and a yellow sphere with a diameter of 2 cm was presented at the end of the tracer. The length of the pole was 20 cm, but its color was white, unlike that in Fig. 1. The diameter of the invisible target trajectory was 30 cm. In this study, the X-, Y-, and Z-axes corresponded to the lateral, vertical, and depth dimensions.

### Experimental procedure

**Figure 2:**
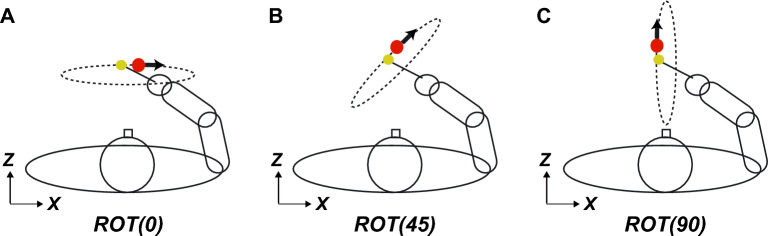
Experimental task. The tracking task in this experiment comprised three kinds of tasks: ROT (0), ROT (45), and ROT (90). For the ROT (0) task, the target moved in a circle on a fronto-parallel plane. For the ROT (45) task, the target moved in a circle on the plane rotated by 45° with respect to the sagittal plane. For the ROT (90) task, the target moved in a circle on the sagittal plane. Participants were instructed to track the target moving at a constant speed of 0.5 Hz.

In this study, we designed an experiment to investigate the motor control mechanism during a tracking task in 3D VR space. As shown in Fig. 2, the experiment involved three kinds of tasks with different depth planes: ROT (0), in which the target was presented by moving in a circle on a fronto-parallel plane; ROT (45), in which the target moved in a circle on a plane rotated by 45° with respect to the sagittal plane; and ROT (90), in which the target moved in a circle on the sagittal plane. In other words, the ROT (0) task required lateral and vertical movements, whereas the ROT (90) task required vertical and depth movements. In contrast, ROT (45) required concurrent lateral, vertical, and depth movements. In addition, this experiment comprised two vision conditions: binocular vision condition, where participants performed the tasks without having their vision blocked in any way, and monocular vision condition, where participants performed the tasks with one side of their vision obstructed.

Before the experiment, the participant sat on a chair and wore the HMD, followed by a calibration session in which the individualized initial position of the target was varied across the height of each participant. The arm length was set to minimize variations due to the diversity of physical characteristics^28^. The participants manipulated the controller with their dominant hand in all tasks. For the monocular condition, participants wore an eye patch to block one side of their eye, identical to the opposite side of their dominant hand, prior to the experiment. For each trial, a sound was generated three times for 3 s, with an interval of 1 s. After 3 s, the target began to move at a constant tangential velocity (0.5 Hz at which the target moved in a circle in 2 s), following the invisible circle in a clockwise direction, as shown in Figs. 1 and 2. The target was stopped after moving in a circle three times. The participants were instructed to match the center of the tracer to that of the target during the task. There were three trials for each task after each trial, which was discarded from analysis. Each participant conducted 12 trials, including a practice trial for each vision condition.

### Data analysis

During the movement task, we recorded the positions of the target and tracer in 3D space at a 90 Hz sampling rate. To evaluate performance, we calculated the position error in 3D space and the absolute value of the error on each axis, which are defined as follows:

1 $$ error \, in \, 3D\, \left[ {mm} \right] = \sqrt {\left( {T_{x} - t_{x} } \right)^{2} + \left( {T_{y} - t_{y} } \right)^{2} + \left( {T_{z} - t_{z} } \right)^{2} } $$

2 $$ error_{X - axis} \left[ {mm} \right] = \left| {T_{x} - t_{x} } \right| $$

3 $$ error_{Y - axis} \left[ {mm} \right] = \left| {T_{y} - t_{y} } \right| $$

4 $$ error_{Z - axis} \left[ {mm} \right] = \left| {T_{z} - t_{z} } \right| $$

where $T_{x}$, $T_{y}$, and $T_{z}$ represent the X, Y, and Z coordinates of the target position, respectively, and $t_{x}$, $t_{y}$, and $t_{z}$ represent the X, Y, and Z coordinates of the tracer’s position, respectively. We averaged these values over the performance time for the analysis.

For each condition and parameter (the abovementioned errors), we performed a one-way repeated measures analysis of variance (ANOVA) (three levels for the plane factor: *ROT (0)*, *ROT (45)*, and *ROT (90)*) using SPSS Statistics (IBM, Armonk, NY, USA). The sphericity assumption was verified using Mauchly’s test. For Mauchly’s test, *p* < 0.05, the p-value for the ANOVA was corrected using Greenhouse–Geisser. A post-hoc test was conducted through pairwise comparisons using Bonferroni correction. We considered comparisons yielding *p* < 0.05 to be statistically significant and comparisons yielding *p* < 0.005 to be highly statistically significant.

### Ethics declarations

All participants provided written informed consent before the experiment. This study was approved by the Institutional Review Board of National Institute of Technology, Gunma College, and conducted in accordance with the Declaration of Helsinki.

## Results

### Tracking performance in the binocular vision condition

**Figure 3:**
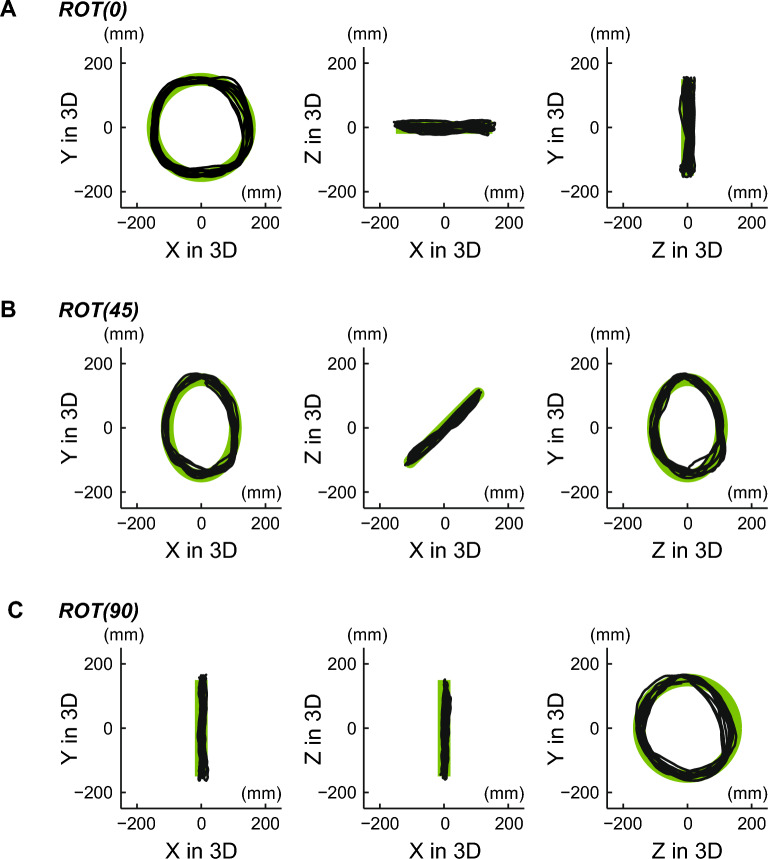
An example of tacking movement in the binocular vision condition. Each row represents the ROT (0), ROT (45), and ROT (90) tasks, respectively. The green line represents the movement of the target moving at a constant speed, and the black line represents the trajectory of the tracer.

**Figure 4:**
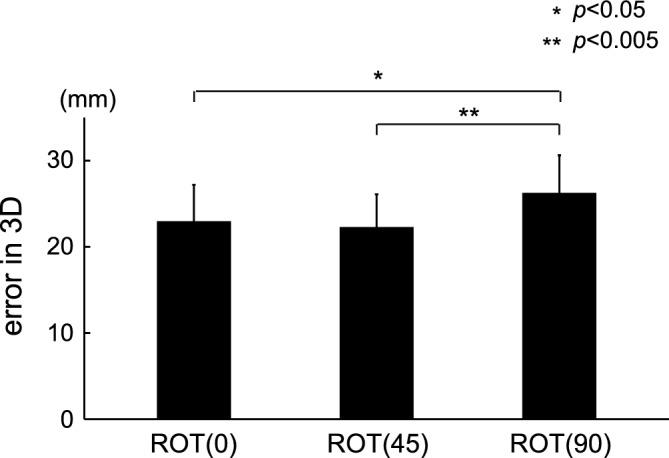
Errors in 3D for the three tasks in the binocular vision condition. We found significant differences in errors between the ROT (0) and ROT (90) tasks and between the ROT (45) and ROT (90) tasks ($${\text{t}}\left( {14} \right) = 3.608$$, corrected $$p = 0.009$$ for the comparison between the ROT (0) and ROT (90) tasks; $${\text{t}}\left( {14} \right) = 4.619$$, corrected $$p = 0.001$$ for the ROT (45) and ROT (90) tasks). The difference in error in 3D between the ROT (0) and ROT (45) tasks was not significant ($${\text{t}}\left( {14} \right) = 0.987$$, corrected $$ p = 1$$).

Figure 3 shows an example of tracking movement in the binocular vision condition. Although certain errors in the depth axis were observed in the ROT (45) and ROT (90) tasks, the tracer generally tracked the target with small errors. Figure 4 shows the 3D errors for all types of tasks in the binocular vision condition. The mean and SD of the errors in 3D were 22.9 $\pm$ 4.3, 22.2 $\pm$ 3.9, and 26.2 $\pm$ 4.4 for ROT (0), ROT (45), and ROT (90), respectively. In the comparison between the ROT (0) and ROT (45) tasks, no significant difference in tracking performance was observed (corrected $p = 1$). However, we observed a significantly large error in 3D in the ROT (90) task (corrected $p = 0.009$ for the comparison between the ROT (0) and ROT (90) tasks; corrected $p = 0.001$ for the ROT (45) and ROT (90) tasks).

**Figure 5:**
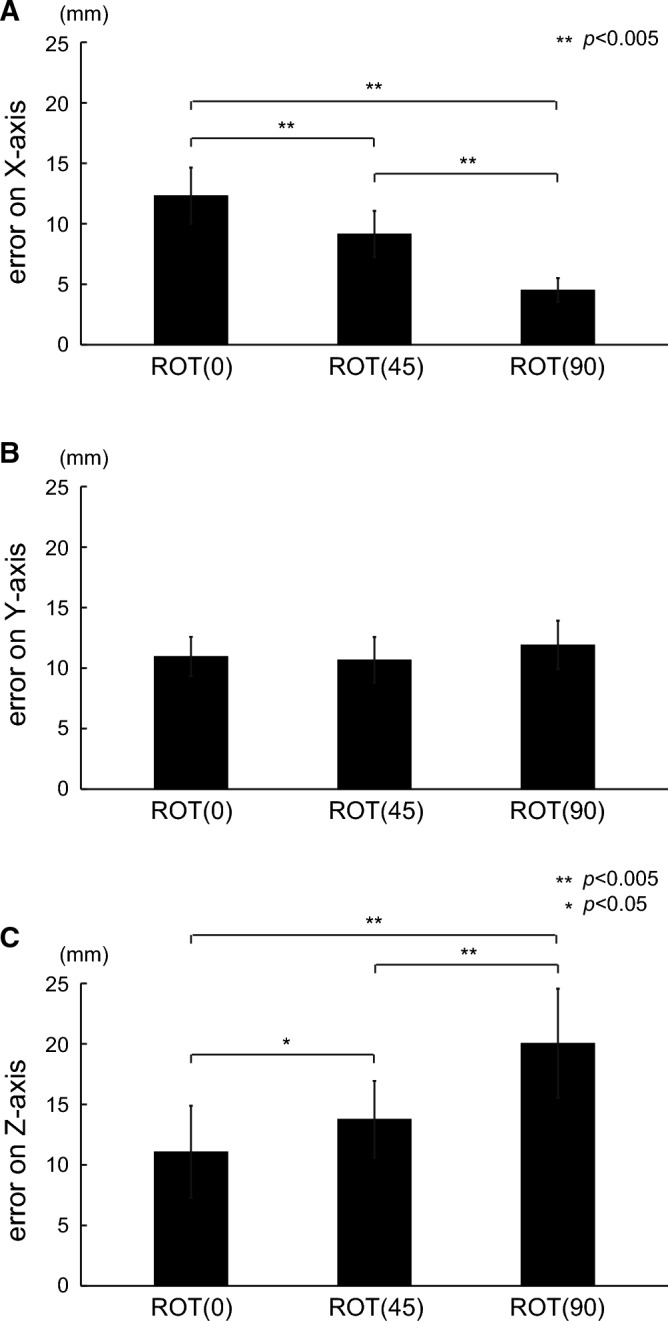
Errors on each axis for the three tasks in the binocular vision condition. Each row represents the X-, Y-, and Z-axis errors, respectively. The differences in errors on the X-axis were significant (${\text{t}}\left( {14} \right) = 7.798$, corrected $p < 0.001$ for the ROT (0) and ROT (45) tasks; ${\text{t}}\left( {14} \right) = 8.235$, corrected $p < 0.001$ for the ROT (45) and ROT (90) tasks; ${\text{t}}\left( {14} \right) = 12.536$, corrected $p < 0.001$ for the ROT (0) and ROT (90) tasks). The differences in errors on the Y-axis were not significant (${\text{F}}\left( {1.283,{ }17.958} \right) = 3.887,{ }p = 0.056$ corrected by Greenhouse–Geisser). For the Z-axis, all the differences in errors were statistically significant (${\text{t}}\left( {14} \right) = 3.117$, corrected $p = 0.023$ for the ROT (0) and ROT (45) tasks; ${\text{t}}\left( {14} \right) = 6.553$, corrected $p < 0.001$ for the ROT (45) and ROT (90) tasks; ${\text{t}}\left( {14} \right) = 8.283$, corrected $p < 0.001$ for the ROT (0) and ROT (90) tasks).

As we found significant differences in the 3D errors, we investigated the errors on each axis. Figure 5 shows the errors on each axis for the three types of tasks in the binocular vision condition. The errors on the X-axis were 12.3 $\pm$ 2.3, 9.15 $\pm$ 1.9, and 4.52 $\pm$ 1.0 for the ROT (0), ROT (45), and ROT (90) tasks, respectively. These differences were significant for all comparisons (corrected $p < 0.001$ for the ROT (0) and ROT (45) tasks; corrected $p < 0.001$ for the ROT (45) and ROT (90) tasks; and corrected $p < 0.001$ for the ROT (0) and ROT (90) tasks). For the Y-axis, the errors were 11.0 $\pm$ 1.6, 10.7 $\pm$ 1.9, and 11.9 $\pm$ 2.0 for the ROT (0), ROT (45), and ROT (90) tasks, respectively. The error in the ROT (90) task was the largest, but not significant, as tested with ANOVA ($p = 0.056$ corrected using Greenhouse–Geisser). For the Z-axis, the errors were 11.1 $\pm$ 3.8, 13.8 $\pm$ 3.2, and 20.1 $\pm$ 4.5 for the ROT (0), ROT (45), and ROT (90) tasks, respectively. Among the tasks, performance in the ROT (90) task was the worst, while performance in the ROT (0) task was the best. The differences were statistically significant (corrected $p = 0.023$ for the ROT (0) and ROT (45) tasks; corrected $p < 0.001$ for the ROT (45) and ROT (90) tasks; and corrected $p < 0.001$ for the ROT (0) and ROT (90) tasks).

### Tracking performance in the monocular vision condition

**Figure 6:**
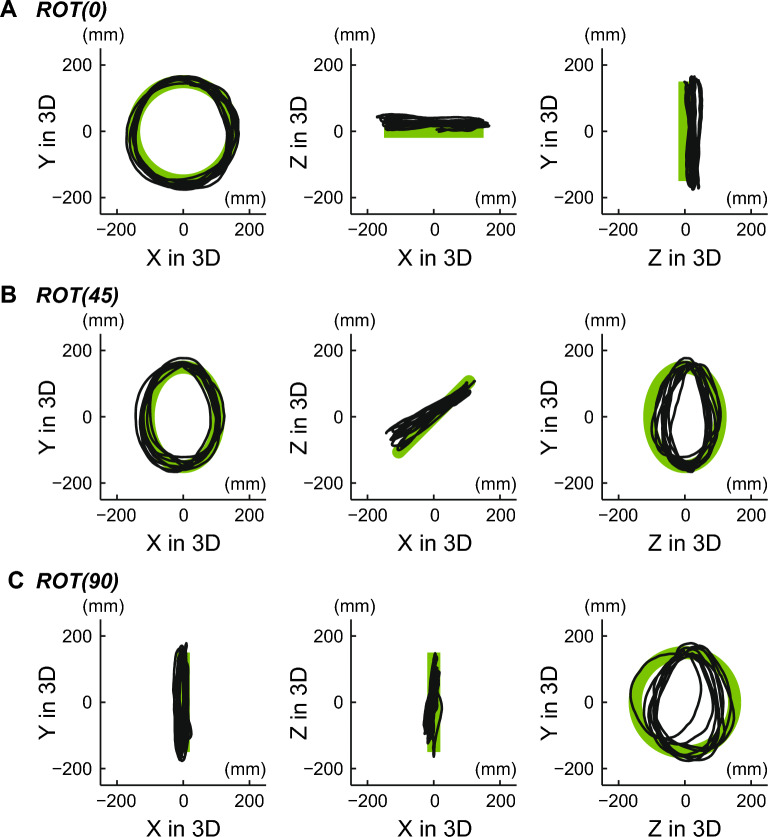
An example of tracking movement in the monocular vision condition. Each row represents the ROT (0), ROT (45), and ROT (90) tasks, respectively. The green line represents the target’s movement at a constant speed, and the black line represents the trajectory of the tracer. The tracer along the depth axis in the ROT (90) task frequently deviated from the correct path.

**Figure 7:**
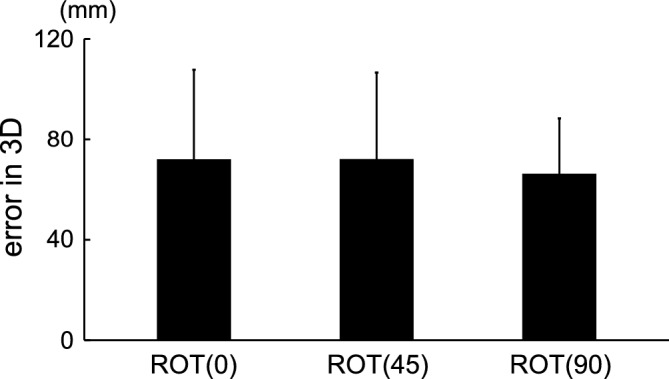
Errors in 3D for the three tasks in the monocular vision condition. None of the differences in errors in 3D were statistically significant (${\text{F}}\left( {2,28} \right) = 0.621,{ }p = 0.545$).

Figure 6 shows an example of tracking movement in the monocular vision condition, indicating that the tracer on the depth axis in the ROT (90) task frequently deviates from the correct path. Figure 7 shows the 3D errors for all types of tasks in the monocular vision condition. The mean and SD of the errors in 3D were 59.6 $\pm$ 34.5, 59.3 $\pm$ 32.9, and 56.4 $\pm$ 21.1 for the ROT (0), ROT (45), and ROT (90) tasks, respectively. The differences in 3D errors for all types of tasks were not statistically significant, as tested with ANOVA (*p* = 0.545).

**Figure 8:**
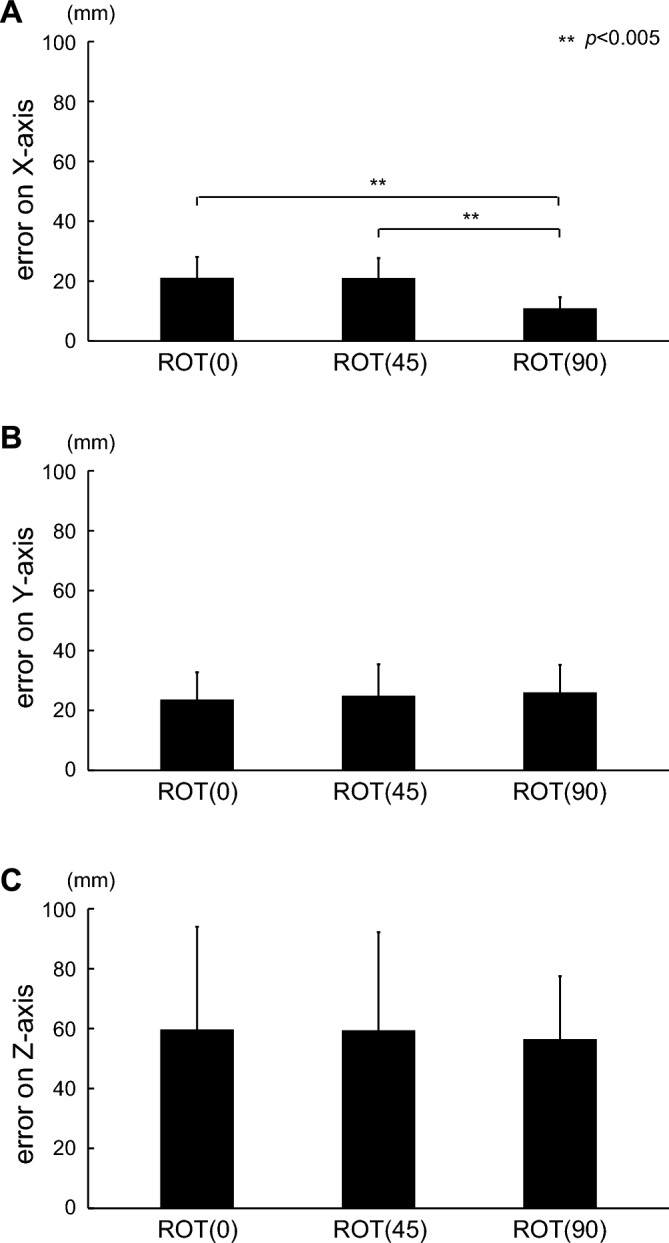
Errors on each axis for the three tasks in the monocular vision condition. Each row represents the X-, Y-, and Z-axis errors, respectively. The error on the X-axis in the ROT (90) task was significantly greater than those in the other tasks (${\text{t}}\left( {14} \right) = 4.554$, corrected ${\text{p}} < 0.001$ for the ROT (0) and ROT (90) tasks; ${\text{t}}\left( {14} \right) = 5.507$, corrected $p < 0.001$ for the ROT (45) and ROT (90) tasks). However, the difference between the ROT (0) and ROT (45) tasks was not significant (${\text{t}}\left( {14} \right) = 0.039$, corrected $p = 1$ for the ROT (0) and ROT (45) tasks). For the errors on the Y-axis, a significant difference among tasks was not observed (${\text{F}}\left( {2,28} \right) = 0.755,{ }p = 0.48$). Similar to the Y-axis, the errors on the Z-axis were the same in all tasks (${\text{F}}\left( {2,28} \right) = 0.197,{ }p = 0.822$).

Figure 8 shows the errors on each axis for the three types of tasks in the monocular vision condition. The errors on the X-axis were 21.0 $\pm$ 7.1, 20.9 $\pm$ 6.8, and 10.8 $\pm$ 3.9 for the ROT (0), ROT (45), and ROT (90) tasks, respectively. The performance in the ROT (90) task was significantly better than that in the other tasks (corrected $p < 0.001$ for the ROT (0) and ROT (90) tasks; corrected $p < 0.001$ for the ROT (45) and ROT (90) tasks). However, the difference between the ROT (0) and ROT (45) tasks was not significant (corrected $p = 1$ for the ROT (0) and ROT (45) tasks, respectively). For the error on the Y-axis, the performance in all tasks was the same as tested with ANOVA (*p* = 0.48). The mean and SD of the errors on the Y-axis were 23.5 $\pm$ 9.3, 24.8 $\pm$ 10.7, and 25.9 $\pm$ 9.1 for the ROT (0), ROT (45), and ROT (90) tasks, respectively. Similar to the Y-axis, the errors on the Z-axis were the same in all tasks, as tested with ANOVA (*p* = 0.822). The errors on the Z-axis were 59.6 $\pm$ 34.5, 59.3 $\pm$ 32.9, and 56.4 $\pm$ 21.1 for the ROT (0), ROT (45), and ROT (90) tasks, respectively.

## Discussion

In this study, we quantitatively evaluated the tracking performance of circular movements using three types of tasks with different depth planes in 3D VR space. In particular, we adopted two vision conditions (binocular and monocular vision) and investigated the effect of the required movement along the depth axis during tracking tasks in 3D VR space. Under binocular vision, the ROT (90) task, which requires the largest depth movement, showed the greatest error in 3D among all tasks (Fig. 4). Likewise, the errors on the depth axis revealed significant differences among the tasks (Fig. 5). Under monocular vision, significant differences in errors were observed only on the x-axis (Fig. 8). These findings indicate that the amount of required depth movement under binocular vision determines the depth error independently of the other axes, implying that the brain may independently process binocular vision information on each axis. Meanwhile, the required depth movement under monocular vision was independent of performance along the depth axis, indicating an intractable behavior.

For the binocular vision condition, the error in 3D in the ROT (90) task was significantly high, suggesting that a task requiring movement on the depth axis is demanding. When we looked at the decomposed errors along each axis, the ROT (0) task involved the largest error on the X-axis, whereas the ROT (90) task involved the largest error on the Z-axis. Errors in the ROT (45) task were between those in the ROT (0) and ROT (90) tasks, regardless of the axis, implying that information on each axis may be independently processed in the brain. In a previous circular tracking task, a significantly different error on the X-axis between the ROT (0) and ROT (90) tasks was reported^28^, in line with our current findings. The errors on the Y-axis were the same, regardless of the rotation. Given that the distance traversed on the Y-axis is the same on all tasks, Y-axis performance is not influenced by information regarding the other axes, supporting the idea that the brain may process information regarding this axis independently (Supplementary Tables).

For the monocular vision condition, errors on the X-axis were involved in the significant difference between the ROT (90) task and either the ROT (0) or ROT (45) task. In addition, the error was not significantly different between the ROT (0) and ROT (45) tasks, suggesting that the amount of horizontal information required to perform the task was not proportional to actual performance. On the depth axis, although no significant differences were observed, the mean error was approximately 60 mm, which is three times greater than the errors in the other axes; this finding was supported by those of a previous study reporting that people tend to underestimate the distance of objects under monocular vision^30,31^. The insignificant differences were attributed to this high variance. Thus, information processing within the brain under monocular vision is more complicated than that under binocular vision.

This difference between monocular and binocular vision may result from several types of information available only under binocular vision, which might include binocular vision involving a better understanding of the properties of a target object^32^. The advantage of binocular vision is associated with spatial accuracy in completing tasks^33^. Furthermore, its superiority has been reported in several motor tasks involving catching^34^ or walking^35^ as well as when performing tasks that require complex motor skills, such as a handspring^36^ or handling tools^37^, which supports the validation of our results. In addition, we observed a floor effect of monocular vision that caused large errors in the depth axis across all groups, with insignificant differences when performing a tracking task. In a previous study, the floor effect of monocular vision on tracking performance was observed by blurred vision^38^. These might be used for quantifying the other kinds of capabilities of vision that suffer by depriving one side of vision, considering that performance could be maintained by substituting information only available under binocular vision with that available under monocular vision^39^. Our study revealed that binocular vision outperformed monocular vision in basic tracking tasks.

This study observed only behavioral performance. However, several neural activities correlate with depth processing, including V1 for depth processing^40^, V4 for binocular disparity^41^, and V2 for processing relative disparity^42^. Moreover, out of several kinds of stimuli, the disparity stimulus has been reported to induce the strongest response in the human visual cortex^43^, supporting that positional information is extraordinarily important for visual processing and was used in our study. Moreover, when the primary visual cortex was optogenetically suppressed, jump performance deteriorated in both binocular and monocular vision, which is directly linked to the attribution of behavioral performance to neural activity^39^. In addition to the visual cortex, the corpus callosum is associated with midline depth perception^44^. Studies involving non-human primates have also demonstrated the relationship between depth processing and neural response. For instance, the macaque parietal cortex, related to depth processing, was mainly activated during movement execution rather than during preparation^45^. Additionally, movement direction in a planar reaching task, where movement direction determined differing amounts of required movements along the depth axis, was involved in dorsal premotor cell activity^46^, and V6A neurons^47^. Direction on a fronto-parallel plane was also associated with neuronal activities in the premotor or primary motor cortex^48^, inferring that direction is independent of depth processing. Investigations in the PE area in macaques revealed segregated processing of depth and direction even though the area was involved in the processing of both kinds of information^49^. These studies support the idea that our results can be attributed to neural activity.

Several factors should be considered in future studies. Because visual information, including depth information, is most likely to be used more during feedback control than during feedforward control, how depth information is used during tracking movement should be considered. Since tracking performance is mainly governed by feedforward control, and feedback control is sometimes used for error correction^50^, the feedback acts’ frequency, duration, and phase should be investigated. In addition, we adopted a constant target speed, but this speed affects motor control in a tracking task^51,52^. Humans’ general binocular movement is adjusted to the natural environment^53^; therefore, eye movement during the task could also be measured to see how different the required eye movement for a task is from the adjusted eye movement in a future study. It has been reported that the predictability of a target could modify the control strategy in tracking tasks^1,54^. Although these factors make it difficult to investigate tracking movement, as a first step, we found that the brain might process the information on each axis independently under binocular vision. Moreover, the process of error generation may involve more aspects, and may be possible to investigate from biomechanical and physiological standpoints.

In this study, we investigated how behavioral tracking performance would be affected by the required depth information so that we could understand how the brain deals with information on each axis. We observed that the errors in the lateral and depth axes were proportional to the required movement on the lateral and depth axes under binocular vision. The tracking performance was unpredictable based on the required lateral movement under monocular vision. Despite its intricate information-processing mechanisms, we confirmed that depth information under binocular vision was independently processed from that in the other axes, whereas monocular vision involved intractable behavior. Therefore, tracking performance varied in a more non-linear way with the amount of required depth movement. This was contrary to the performance that showed increased depth axis errors and decreased lateral axis errors proportional to required depth movement under the binocular condition.

### Supplementary Information

Supplementary Tables.

## Data Availability

The datasets generated during and/or analyzed during the current study are available from the corresponding author on reasonable request.
