# Supplementary material for: The effect of different depth planes during a manual tracking task in three-dimensional virtual reality space
Source: Sci Rep. 2023 Dec 6;13:21499. doi: 10.1038/s41598-023-48869-w (PMC10700492; doi:10.1038/s41598-023-48869-w)
Supplement: Supplementary file 1 — Supplementary Tables. [file 41598_2023_48869_MOESM1_ESM.docx]

# **Supplementary Tables**

# **Table S1. Individual performance (three-dimensional error). Unit: [mm]**

| Subject | Monocular | | | Binocular | | |
| --- | --- | --- | --- | --- | --- | --- |
|  | ROT (0) | ROT (45) | ROT (90) | ROT (0) | ROT (45) | ROT (90) |
| 1 | 45.00 | 37.48 | 59.67 | 17.25 | 15.74 | 24.12 |
| 2 | 61.71 | 89.68 | 48.88 | 16.09 | 18.38 | 19.08 |
| 3 | 90.92 | 113.08 | 58.12 | 23.99 | 23.12 | 23.16 |
| 4 | 136.51 | 148.93 | 94.62 | 29.13 | 27.44 | 27.23 |
| 5 | 44.94 | 42.39 | 42.02 | 21.47 | 22.87 | 21.78 |
| 6 | 54.62 | 39.56 | 54.54 | 18.51 | 23.01 | 30.32 |
| 7 | 96.23 | 67.14 | 58.68 | 20.53 | 21.83 | 23.22 |
| 8 | 95.86 | 87.12 | 61.84 | 26.43 | 26.18 | 28.17 |
| 9 | 41.04 | 69.22 | 52.03 | 29.99 | 27.21 | 34.81 |
| 10 | 31.00 | 25.42 | 48.57 | 25.43 | 22.15 | 25.94 |
| 11 | 108.09 | 104.24 | 97.80 | 23.80 | 19.45 | 25.59 |
| 12 | 132.46 | 91.93 | 118.43 | 27.23 | 23.83 | 32.45 |
| 13 | 32.91 | 34.03 | 45.56 | 19.38 | 18.83 | 24.30 |
| 14 | 71.45 | 76.77 | 82.46 | 24.30 | 27.23 | 30.95 |
| 15 | 35.99 | 52.63 | 68.02 | 20.42 | 16.31 | 22.05 |
| Mean | 71.92 | 71.98 | 66.08 | 22.93 | 22.24 | 26.21 |
| SD | 35.82 | 34.58 | 22.27 | 4.26 | 3.86 | 4.41 |

SD, standard deviation; ROT (0), target moved circular in the fronto-parallel plane; ROT (45), target moved circular rotated by 45° in the sagittal plane; ROT (90), target moved circular in the sagittal plane.

# **Table S2. Individual performance (X-axis error). Unit: [mm]**

| Subject | Monocular | | | Binocular | | |
| --- | --- | --- | --- | --- | --- | --- |
|  | ROT (0) | ROT (45) | ROT (90) | ROT (0) | ROT (45) | ROT (90) |
| 1 | 16.67 | 16.28 | 13.50 | 9.97 | 7.84 | 4.14 |
| 2 | 14.73 | 17.01 | 7.27 | 9.48 | 6.42 | 2.98 |
| 3 | 17.99 | 23.37 | 7.35 | 12.89 | 7.22 | 6.87 |
| 4 | 31.49 | 32.50 | 10.67 | 14.61 | 9.53 | 4.42 |
| 5 | 16.50 | 14.33 | 9.79 | 11.38 | 10.26 | 5.39 |
| 6 | 16.94 | 17.05 | 12.18 | 9.95 | 7.66 | 4.25 |
| 7 | 23.39 | 16.23 | 9.60 | 10.72 | 8.90 | 3.81 |
| 8 | 31.09 | 24.09 | 8.25 | 12.85 | 10.92 | 5.22 |
| 9 | 16.99 | 25.59 | 11.63 | 15.14 | 9.34 | 4.97 |
| 10 | 15.58 | 10.62 | 8.39 | 11.72 | 8.73 | 4.10 |
| 11 | 26.26 | 27.01 | 11.07 | 11.48 | 8.30 | 3.22 |
| 12 | 32.55 | 22.01 | 9.32 | 16.23 | 10.62 | 4.05 |
| 13 | 10.50 | 11.50 | 9.28 | 10.33 | 8.31 | 5.00 |
| 14 | 27.56 | 31.33 | 10.30 | 16.57 | 14.41 | 3.83 |
| 15 | 15.95 | 24.43 | 23.18 | 11.53 | 8.80 | 5.47 |
| Mean | 20.95 | 20.89 | 10.79 | 12.32 | 9.15 | 4.51 |
| SD | 7.11 | 6.78 | 3.85 | 2.32 | 1.91 | 0.99 |

SD, standard deviation; ROT (0), target moved circular in the fronto-parallel plane; ROT (45), target moved circular rotated by 45° in the sagittal plane; ROT (90), target moved circular in the sagittal plane.

# **Table S3. Individual performance (Y-axis error). Unit: [mm]**

| Subject | Monocular | | | Binocular | | |
| --- | --- | --- | --- | --- | --- | --- |
|  | ROT (0) | ROT (45) | ROT (90) | ROT (0) | ROT (45) | ROT (90) |
| 1 | 22.20 | 18.81 | 33.06 | 8.05 | 7.58 | 14.03 |
| 2 | 21.45 | 27.31 | 18.49 | 9.44 | 8.52 | 10.16 |
| 3 | 25.13 | 29.31 | 18.58 | 10.87 | 9.69 | 10.11 |
| 4 | 40.25 | 51.21 | 33.17 | 13.09 | 12.52 | 13.72 |
| 5 | 15.80 | 16.21 | 17.75 | 11.86 | 11.58 | 10.66 |
| 6 | 17.72 | 14.98 | 21.88 | 9.79 | 10.85 | 14.85 |
| 7 | 29.52 | 22.02 | 21.35 | 11.12 | 11.02 | 9.64 |
| 8 | 30.40 | 27.45 | 25.18 | 13.00 | 14.50 | 14.57 |
| 9 | 15.84 | 23.84 | 19.04 | 13.07 | 11.42 | 13.51 |
| 10 | 15.20 | 12.58 | 23.67 | 12.74 | 11.71 | 11.57 |
| 11 | 29.35 | 34.15 | 33.58 | 9.48 | 9.92 | 10.61 |
| 12 | 41.01 | 36.24 | 44.31 | 10.54 | 11.02 | 13.32 |
| 13 | 13.51 | 14.30 | 16.95 | 9.82 | 9.58 | 11.02 |
| 14 | 24.32 | 30.01 | 43.38 | 12.11 | 12.56 | 12.44 |
| 15 | 10.66 | 12.82 | 17.49 | 9.31 | 7.66 | 8.37 |
| Mean | 23.49 | 24.75 | 25.86 | 10.95 | 10.67 | 11.91 |
| SD | 9.26 | 10.67 | 9.35 | 1.63 | 1.90 | 2.01 |

SD, standard deviation; ROT (0), target moved circular in the fronto-parallel plane; ROT (45), target moved circular rotated by 45° in the sagittal plane; ROT (90), target moved circular in the sagittal plane.

# **Table S4. Individual performance (Z-axis error). Unit: [mm]**

| Subject | Monocular | | | Binocular | | |
| --- | --- | --- | --- | --- | --- | --- |
|  | ROT (0) | ROT (45) | ROT (90) | ROT (0) | ROT (45) | ROT (90) |
| 1 | 31.33 | 23.35 | 43.36 | 7.93 | 8.49 | 15.45 |
| 2 | 52.02 | 80.28 | 42.08 | 5.12 | 12.39 | 13.09 |
| 3 | 81.98 | 102.54 | 52.69 | 12.82 | 16.67 | 16.69 |
| 4 | 122.09 | 129.38 | 84.93 | 16.13 | 19.03 | 20.11 |
| 5 | 34.69 | 32.07 | 34.40 | 8.55 | 13.05 | 15.26 |
| 6 | 45.20 | 28.71 | 46.17 | 8.05 | 15.61 | 23.53 |
| 7 | 85.31 | 56.86 | 51.46 | 8.54 | 13.38 | 18.32 |
| 8 | 78.66 | 73.72 | 52.52 | 14.31 | 14.18 | 20.56 |
| 9 | 29.21 | 55.51 | 43.83 | 16.96 | 19.15 | 29.49 |
| 10 | 17.73 | 15.13 | 37.62 | 14.91 | 13.74 | 20.67 |
| 11 | 96.71 | 90.47 | 89.03 | 14.55 | 11.35 | 20.86 |
| 12 | 115.12 | 77.57 | 106.32 | 13.67 | 15.12 | 26.02 |
| 13 | 24.05 | 25.02 | 39.01 | 8.42 | 11.31 | 18.52 |
| 14 | 52.43 | 56.47 | 62.65 | 6.70 | 14.51 | 25.27 |
| 15 | 27.06 | 42.18 | 59.44 | 9.43 | 8.51 | 16.91 |
| Mean | 59.57 | 59.28 | 56.37 | 11.07 | 13.77 | 20.05 |
| SD | 34.46 | 32.90 | 21.13 | 3.81 | 3.17 | 4.51 |

SD, standard deviation; ROT (0), target moved circular in the fronto-parallel plane; ROT (45), target moved circular rotated by 45° in the sagittal plane; ROT (90), target moved circular in the sagittal plane.
